# Application of the Three-Group Model to the 2024 US Elections

**DOI:** 10.3390/e27090935

**Published:** 2025-09-06

**Authors:** Miron Kaufman, Sanda Kaufman, Hung T. Diep

**Affiliations:** 1 Department of Physics, Cleveland State University, Cleveland, OH 44115, USA; 2 Levin School of Urban Affairs, Cleveland State University, Cleveland, OH 44115, USA; s.kaufman@csuohio.edu; 3 Laboratoire de Physique Théorique et Modélisation, Institut Sciences et Techniques, CY Cergy Paris Université, CNRS, UMR 8089, 2, Avenue Adolphe Chauvin, 95302 Cergy-Pontoise Cedex, France; diep@cyu.fr

**Keywords:** political polarization, dynamic modeling, anticipatory scenarios, opinion dynamics, voting, statistical physics approaches for social dynamics

## Abstract

Political polarization in Western democracies has accelerated in the last decade, with negative social consequences. Research across disciplines on antecedents, manifestations and societal impacts is hindered by social systems’ complexity: their constant flux impedes tracing causes of observed trends and prediction of consequences, hampering their mitigation. Social physics models exploit a characteristic of complex systems: what seems chaotic at one observation level may exhibit patterns at a higher level. Therefore, dynamic modeling of complex systems allows anticipation of possible events. We use this approach to anticipate 2024 US election results. We consider the highly polarized Democrats and Republicans, and Independents fluctuating between them. We generate average group-stance scenarios in time and explore how polarization and depolarization might have affected 2024 voting outcomes. We find that reducing polarization might advantage the larger voting group. We also explore ways to reduce polarization, and their potential effects on election results. The results inform regarding the perils of polarization trends, and on possibilities of changing course.

## 1. Introduction

**Figure 1:**
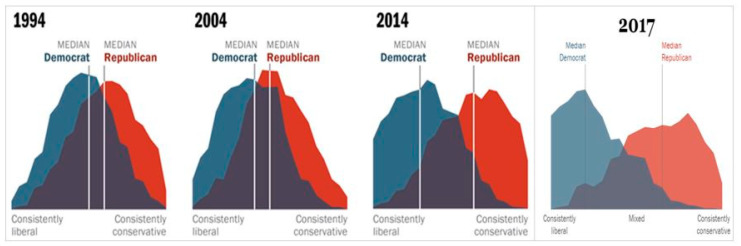
Distribution of Democrats and Republicans on a 10-item scale of political values (Pew Research Center 1994–2014 & 2017).

Political polarization consists of intense clustering of attitudes [1]. While not recent [2], it is rising in the United States and elsewhere. The Pew Research Center surveys [3,4,5] show the US “liberal–conservative” split increasingly widening in the 21st century (Figure 1). Democrat/Republican political affiliation has become defining, both professionally and socially, discouraging interpersonal and inter-group dialogues [6,7,8,9] which are key in negotiations and joint decision making. As ref. [10] observed, “Rising political polarization in recent decades has hampered and gridlocked policymaking, as well as weakened trust in democratic institutions.”

Polarization is in a chicken-and-egg relationship with homophily [11]. The latter is individuals’ preference to engage only with members of their own social/political group. Polarization and homophily enhance each other, with negative societal consequences [12]. Together, they have led to disjointed factual bases that have diminished public trust in science [13] and in media and governments [14], which had begun before COVID [15]. The COVID crisis fueled both opinion differences and homophily through increased social isolation due to lack of direct communication, and to growing reliance on social media. In turn, traditional and social media exacerbate polarization by reducing complex situations and people in Manichaean fashion to “good” and “bad” [16]. Thus, information producers and consumers on social media platforms, and the platforms themselves, are now split by political identity.

Polarization may be a consequence of open interactions among a democratic society’s members [17], and—counterintuitively—may even be helpful to democracy in small doses [9]. However, ref. [18] observed that “Extreme polarization can undermine democracy by making compromise impossible and transforming politics into a zero-sum game.” That negotiation—accommodating different interests through mutually beneficial tradeoffs—is at great disadvantage in polarized times, in the absence of common ground on almost any public issue [19]. Across scales, from interpersonal to family, to various local and national groups, and internationally, removing opponents from discourse is increasingly preferred to engagement.

Given the downsides of polarization, scholars have intensified their research [20], from its measurement [4,21,22,23] to social consequences [12], and to remedies [12,24,25]. There is also a strong effort across disciplines and methodologies to model polarization and anticipate trends [7,19,26,27,28], some using scenarios [29,30].

Obstacles to the study of polarization derive from its emergence in time in complex social systems that shift, impeding “all else being equal” research approaches and the tracing of observed effects to their causes. According to [30], “they [polarization trends] are inherently systems-level phenomena, involving interactions among multiple component parts and the emergence of broader-scale features; yet, they have been inadequately explored from that perspective.” Therefore, using social physics tools, scholars have turned to dynamic network models [31], which are anticipatory, rather than explanatory or predictive [24,25,29,32,33].

Sociophysics models rely on a property of complex systems: at “eye level,” the variables necessary to describe a complex system—with many decision-making agents—are numerous and difficult-to-impossible to quantify. However, at “drone level,” complex systems exhibit patterns, which can be described with great economy of variables (compared to “eye level”). The patterns are used to generate scenarios anticipating systems’ trajectories in time under different conditions, deriving ranges of possible outcomes. Probability density modelling affords a macroscopic description, while allowing for microscopic interactions between individuals. We can appeal to the mean-field approximation, from statistical physics, to describe ensemble dynamics under simplifying assumptions about interactions among individuals. We adopt this approach to examine effects of political polarization on the 2024 US national elections. Specifically, we explore whether the results could have been anticipated, how voting outcomes were affected by polarization, and whether it can be reduced (for instance, by leadership).

In this article, we use the sociophysics model proposed by [24] and by [25] to anticipate 2024 US election outcomes in a context of high polarization. We begin by describing this model. We generate a scenario without any intervention, in which voters stick to their positions. Then we produce four illustrative anticipatory scenarios, to explore effects of trying to nudge voters toward the center positions. We discuss the meaning of the scenarios, and the insights they offer for strategizing in future elections. We conclude with some suggestions for how polarization might be countered to mitigate its negative consequences.

## 2. Model

The model pits, in time, the three main voting groups in the US: Democrats, group 1; Republicans, group 2; and Independents, group 3. Each voter has a stance s on a set of issues that are central in the political debates preceding elections. These stances range from extreme adherence to own group preferences, to openness to interaction and dialogue with opponents. Stances s vary between −1 and +1, where −1 corresponds to the Democrats’ most progressive/left position, +1 corresponds to the Republicans’ most conservative/right position and 0 corresponds to the middle of the road/compromise position.

Voters within each group engage with every other of their in-group peers with intensities J1, J2 and J3, respectively, where Democrats are group 1, Republicans are group 2, and independents are group 3. These couplings J quantify the cohesiveness of each group. For Independents, J3 = 0 since they are not identified with a party as a group, and are not interacting in an organized fashion. Democrats and Republicans tend to be homophilic—they engage mostly with others within their own group.

With intensities K, voters also consider the average group stances of their opponents’ opinions. Thus, the model has three intra-group parameters J, and six inter-group parameters K, which we estimate using opinion polls [24]. To explore ways of influencing polarization, we include two fields H and D, which act on individuals’ stances. Field D, corresponding in Physics to the Blume–Capel crystal field [25], “pushes” group stances toward the center, thereby countering polarization; field H enhances homophily by “pushing” individuals toward their own groups. The numerical results shown below correspond to H = 0.

Using the Bolzmann probability weight, we gauge at any time each group’s internal distribution of stances. We compute the average stance of each group using the Boltzmann probability distribution $e^{-\frac{E}{T}}$. The neg-energy associated with an individual in group 1 is:

$$-E1=\left(J1s1+K12s2+K13s3+H1\right)s-D1s^{2},$$

where s is the stance of the individual, and s1, s2, s3, are the mean stances of groups 1, 2, 3, respectively. The fields H1 and D1 represent the action of the leaders on individuals of group 1. For H1>0, the mean stance s1 is pushed towards positive values, while for H1<0 the mean stance is pushed to negative values. The field D1, when positive, favors depolarization: s~0, while when negative it favors polarization: $\left|s\right|\sim 1$. Similarly:

$$-E2=\left(J2s2+K21s1+K23s3+H2\right)s-D2s^{2},\;\mathrm{and}$$

$$-E3=\left(J3s3+K32s2+K31s1+H3\right)s-D3s^{2}.$$

The means are calculated using the distribution functions defined next.

In the mean-field approximation, the distribution of individuals in each group among the stances s at time t are:

(1) $$\rho 1\left(s,t\right)=\frac{e^{\left(j1{s1}_{t}+k12{s2}_{t}+k13{s3}_{t}+h1\right)s-d1s^{2}}}{\int_{-1}^{1}e^{\left(j1{s1}_{t}+k12{s2}_{t}+k13{s3}_{t}+h1\right)s-d1s^{2}}ds}\;\rho 2\left(s,t\right)\frac{e^{\left(j2{s2}_{t}+k21{s1}_{t}+k23{s3}_{t}+h2\right)s-d2s^{2}}}{\int_{-1}^{1}e^{\left(j2{s2}_{t}+k21{s1}_{t}+k23{s3}_{t}+h2\right)s-d2s^{2}}ds}\;\rho 3\left(s,t\right)=\frac{e^{\left(j3{s3}_{t}+k31{s1}_{t}+k32{s2}_{t}+h3\right)s-d3s^{2}}}{\int_{-1}^{1}e^{\left(j3{s3}_{t}+k31{s1}_{t}+k32{s2}_{t}+h3\right)s-d3s^{2}}ds}$$

$\rho i\left(s,t\right)ds$ gives the fraction of all individuals in group i who have a stance in the interval (s,s+ds). Lower case parameters j, k, etc., are the uppercase parameters J, K, etc., divided by temperature T. Finally, ${sj}_{t}$ is the group j average at time t.

Our model implementation uses the distributions of attitudes in each group to estimate the vote pattern. We assume that voters with −1<s<−1/3 vote for the Democrat candidate, voters with −1/3<s<1/3 do not vote, and voters with 1/3<s<1 vote for the Republican candidate. The respective sizes of each group are estimated using information (Since the numbers of Democrat and Republican registered voters change in time, we use contemporaneous 2024 figures to anticipate election outcomes from USAFacts.org, published on 30 September 2024) about the party affiliation of registered voters: 45.1 million Democrats, 36 million Republicans, and 32.1 million Independents. We denote by X1, X2, and X3 the fraction of potential voters belonging to each of the three groups: X1 = 0.398, X2 = 0.318, X3 = 0.284. We calculate the fraction of potential voters V1 who vote for the Democrat candidate, V2 for the Republican candidate, and V3 those who do not vote (Note that: Independents may end up voting for either the Democrat or the Republican candidate; and, voters in any of the three groups may end up not voting in the elections):

(2) $$\begin{array}{l} V1\left(t\right)=X1*\int_{-1}^{-1/3}\rho 1\left(s,t\right)ds+X2*\int_{-1}^{-1/3}\rho 2\left(s,t\right)ds+X3*\int_{-1}^{-1/3}\rho 3\left(s,t\right)ds\\ V2\left(t\right)=X1*\int_{1/3}^{1}\rho 1\left(s,t\right)ds+X2*\int_{1/3}^{1}\rho 2\left(s,t\right)ds+X3*\int_{1/3}^{1}\rho 3\left(s,t\right)ds\\ V3\left(t\right)=X1*\int_{-1/3}^{1/3}\rho 1\left(s,t\right)ds+X2*\int_{-1/3}^{1/3}\rho 2\left(s,t\right)ds+X3*\int_{-1/3}^{1/3}\rho 3\left(s,t\right)ds \end{array}$$

To obtain the time evolution of distributions used in Equation (2) above we employ the mean-field approximation. We start by defining the Langevin–Blume-Capel (LBC) function:

(3) $$LBC(h,d)\;=\;\frac{\int_{-1}^{1}se^{hs-ds^{2}}ds}{\int_{-1}^{1}e^{hs-ds^{2}}ds}$$

The variable h in Equation (3) stands for the magnetic field normalized by temperature, which is the sum of the external field and the mean-field generated by in-group interactions ~Js and by intra-group interactions ~Ks. The variable d in Equation (3) stands for the field coupled to s^2^. The ensemble average stances ${s1}_{t+1},\;{s2}_{t+1},\;{s3}_{t+1}$ at time t + 1 are assumed to be determined by preferences of each group at an earlier time t. This lag represents the time it takes to change individuals’ stance. The time t is expressed in units of the lag time. Thus, for each of the three groups respectively:

(4) $$\begin{array}{l} {s1}_{t+1}=LBC(h1+j1{s1}_{t}+k12{s2}_{t}+k13{s3}_{t},d1)\\ {s2}_{t+1}=LBC(h2+j2{s2}_{t}+k21{s1}_{t}+k23{s3}_{t},d2)\\ {s3}_{t+1}=LBC(h3+j3{s3}_{t}+k31{s1}_{t}+k32{s2}_{t},d3) \end{array}$$

where $h1=\frac{H1}{T}$, $k12=\frac{K12}{T}$, $d1=\frac{D1}{T}$, etc. The inter-group interactions K12 and K21 are not necessarily equal. For example, members of one group may feel cooperative toward another group, who might not reciprocate. We use numerical integration to evaluate the LBC function. At each moment in time, we compute a corresponding polarization index, which consists of the difference between Republican and Democrat voting group average stances [24,25] at that time:

P = (s2 − s1)/2. −1 ≤ P ≤ 1(5)

The un-polarized case P = 0 corresponds to equal stances s1=s2. Polarization is extreme when P = 1, which corresponds to the Republicans’ average stance s2=1 (conservative/right) and the Democrats’ average stance s1=−1 (progressive/left); or, when P = −1 corresponding to Republicans’ stance s2=−1 and Democrats’ stance s1=1.

To recapitulate: we solve Equation (4) to generate the group means time dependence; we use them to evaluate the distributions of stances from Equation (1); using the distributions we determine the voting pattern using Equation (2) and the polarization using Equation (5).

## 3. Numerical Results and Discussion

We anticipate vote outcomes using the values of J, K parameters proposed in Kaufman et al., 2022 [24]. The Democrats (group 1) are more cohesive than Republicans (group 2), i.e., J1 > J2; the Independents (group 3) have no cohesion (J3 = 0) because they have no means of identifying with each other; they exert no influence on the other two groups: K13 = K23 = 0; the Independents are contrarian to the party in power (group 1), so K31 < 0, and are not influenced by the opposition party, K32 = 0. The group parameter values chosen are consistent with those assumptions: J1 = 5; J2 = 3; J3 = 0; K12 = −4; K21 = −5; K31 = −3; K13 = K23 = K32 = 0. The numerical results shown in this paper are obtained for T = 1.

We simulate the voting patterns under 5 scenarios. For each scenario we show 3 graphs: a. polarization vs time; b. fractions of potential voters in time voting for the candidate of each of the two parties, and of non-voters; and c. the fraction of voters for each candidate out of all voters vs time. In scenario 1, we apply no intervention, meaning that D1 = D2 = 0. In Scenario 2, we explore the vote pattern when a depolarization field D acts on both Democrat and Republican groups. In scenarios 3 and 4 we apply the depolarization field D on one group only. In scenario 5 we consider the intergroup interactions K12 and K21 to have different signs.

**Figure 2:**
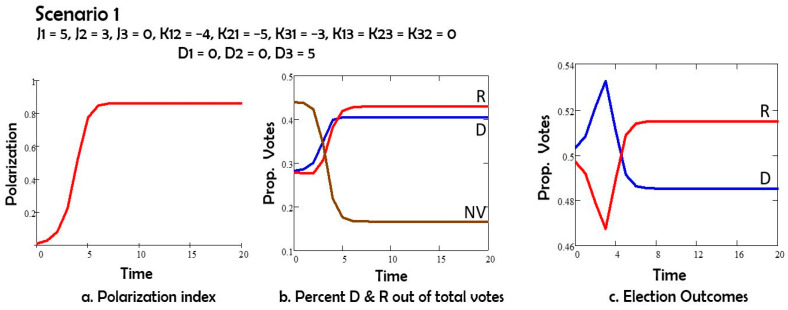
(**a**) Polarization vs time; (**b**) percent of potential voters Democrats D, Republican R, and nonvotes NV vs. time; (**c**) percent of all votes D and R vs. time.

Scenario 1 (Figure 2) shows the time evolution of polarization among the stances of Democrat (D) and Republican (R) voters in a.; the corresponding proportions of D voters, R voters and non-voters (NV) vs. time in b. Figure 2c shows the anticipated voting outcome in time, until the actual vote. The outcome closely corresponds to the actual election result (Source: Wikipedia 2024 United States presidential election, last visited on 25 May 2025. https://en.wikipedia.org/wiki/2024_United_States_presidential_election) in 2024: the Democrat candidate obtained 0.483 of the vote, and the Republican candidate got 0.498 of the vote. If we only consider the two main parties’ candidates (Democrat and Republican), these numbers become: 0.492 and 0.508. The model predictions are quite close: 0.486 and 0.514.

**Figure 3:**
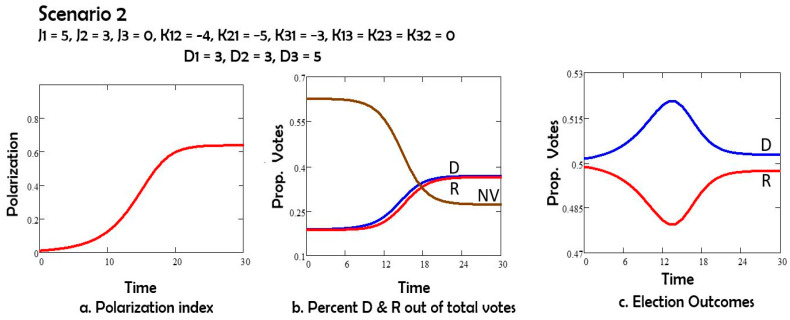
(**a**) Polarization vs time; (**b**) percent of potential voters Democrats D, Republican R, and nonvotes NV vs. time; (**c**) percent of all votes D and R vs. time.

In Scenario 2 (Figure 3), we explore the influence of the de-polarization field D, by assuming that it is the same for both parties: D1 = D2 = 3, nudging voters toward the center. In this scenario, the Democrat candidate wins the election.

**Figure 4:**
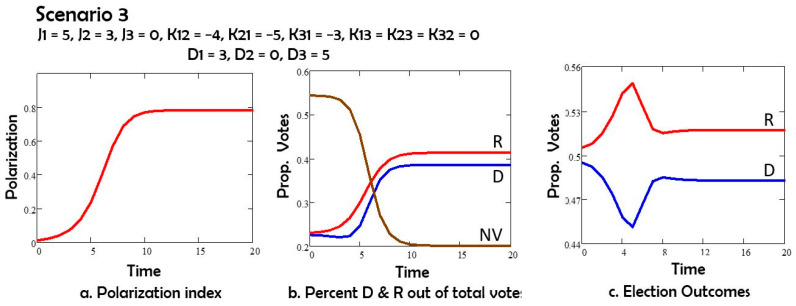
(**a**) Polarization vs time; (**b**) percent of potential voters Democrats D, Republican R, and nonvotes NV vs. time; (**c**) percent of all votes D and R vs. time.

In Scenario 3 (Figure 4) we consider that the depolarization field D acts on Democrats but not on Republicans: D1 = 3, D2 = 0. The Republicans win the election under this assumption.

**Figure 5:**
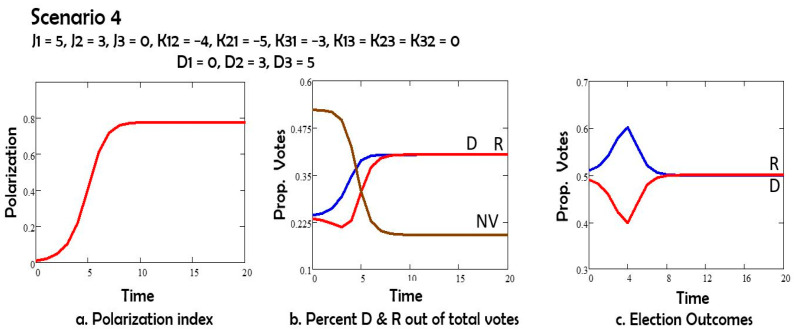
(**a**) Polarization vs time; (**b**) percent of potential voters Democrats D, Republican R, and nonvotes NV vs. time; (**c**) percent of all votes D and R vs. time.

In Scenario 4 (Figure 5) the depolarization field acts on Republicans, but not on Democrats: D1 = 0, D2 = 3. The Democrats win the election under this assumption.

**Figure 6:**
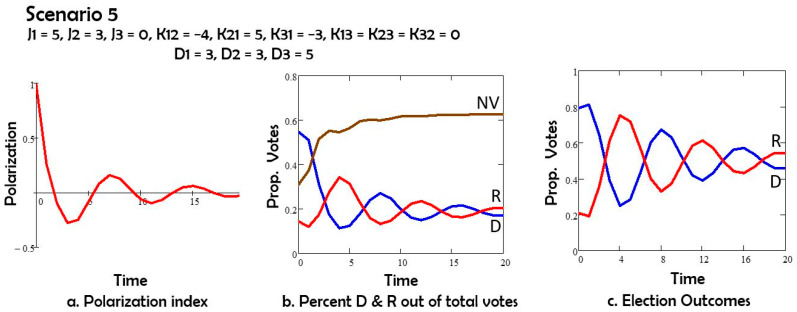
(**a**) Polarization vs time; (**b**) percent of potential voters Democrats D, Republican R, and nonvotes NV vs. time; (**c**) percent of all votes D and R vs. time.

In Scenario 5 (Figure 6) we consider that K21 and K12, the interactions between Democrats and Republicans, have opposite signs. As a result, time oscillations emerge. Then the election result depends on the date at which the voting occurs.

Comparing Scenarios 1 and 2, we note that the depolarization field D acting on both groups 1 (Democrats) and 2 (Republicans) has the effect of reducing polarization, and also of increasing the number of nonvoters. If the depolarization field acts only on group 1 or only on group 2, as in Scenarios 3 and 4, the polarization is still reduced, and the non-voters group still increases in size by comparison to Scenario 1. However, both changes are less than in Scenario 2. The Republicans (group 2) win the elections in Scenarios 1, 3 and 4. For Scenario 5 with oscillations the election results depend on the day of the election.

## 4. Concluding Remarks

We explored five US presidential election scenarios which illustrate the potential of the three-group model to study polarization effects and their mitigation. In the first four scenarios, all interaction parameters J and K within and between political groups remain the same. In the last four scenarios, we introduce intervention to explore the potential for reducing polarization—which we measure with a polarization index—and effects on election outcomes.

In Scenario 1, with no intervention, the Republicans’ presidential candidate wins (we know in retrospect that this was the real outcome);

In Scenario 2, we applied a field D on both Republicans’ and Democrats’ stances, which pushed both groups toward the center. In this case, the Democrats win the elections (Figure 2b). This scenario has, for long times, the lowest polarization index (Figure 2a) when compared to scenarios 1, 3, 4;

In Scenario 3, we applied the D field only on Democrats, nudging them to the center. In this case, Republicans win;

In Scenario 4, the field D only pushed Republicans to the mid stance. Republican and Democrat votes converged, becoming indistinguishable. Democrats and Republicans are equally likely to win.

In Scenario 5, we applied the field D to both Republicans and Democrats, as in Scenario 2. However, unlike in the other scenarios, we altered the way in which the two groups relate to each other: K12 and K21 have opposite signs. As a result, we obtained oscillations, meaning that the election outcome depends on the moment in time at which elections occur. In future work, we will explore the role of the interval length of the attitudes on how the individuals vote or not vote.

We find that with this qualitative model, calibrated using opinion polls, we can anticipate the actual election outcome of 2024 (Scenario 1) rather precisely, although it was by no means expected; for example, ref. [34]’s dynamic forecast in September 2024 predicted the opposite. We also find that depolarization efforts may have surprising results that are context-dependent, in the sense that the total number of actual voters and the respective proportions of Democrat and Republican voters out of the total (which varies in time) matters. Lastly, the scenarios may suggest for each party some strategies of increasing their likelihood of winning.

In the context of agent-based models, ref. [35] observed a general quandary: using simple models comes at the cost of realism, while complicated ones become data-intensive and unwieldy. The qualitative model we proposed is relatively simple, yet it served both to anticipate US elections results and to explore intervention possibilities. In future work, we plan to compare our physical model predictions to social research findings. Furthermore, we will expand the current mean-field study to study the range of interactions by performing Monte Carlo simulations with thermal noise.

## Data Availability

The original contributions presented in this study are included in the article. Further inquiries can be directed to the corresponding author.
